# It’s time to step it up. Why safety investigations in healthcare should look more to safety science

**DOI:** 10.1093/intqhc/mzaa013

**Published:** 2020-03-18

**Authors:** Siri Wiig, Jeffrey Braithwaite, Robyn Clay-Williams

**Affiliations:** 1 SHARE-Centre for Resilience in Healthcare, University of Stavanger, Kjell Arholms gate 41, 4036 Stavanger, Norway; 2 Australian Institute of Health Innovation, Macquarie University, Balaclava Rd, 2109 Sydney, Australia

**Keywords:** investigation, safety science, accident models

## Abstract

Accident models and theoretical foundations underpinning safety investigations are key to understanding how investigators construct causality and make recommendations. Safety science has devoted large efforts to investigating and theorizing about accidents. Why doesn’t healthcare pay more interest to these theories when investigating healthcare accidents? We use established accident theories to suggest how these can support safety investigations in healthcare and provide new lenses to investigatory bodies. We reflect on examples from research and practice in healthcare systems and other high-risk industries. Investigation processes and reports serve multiple purposes. We argue there is an untapped improvement potential for healthcare safety investigations and suggest new ways of integrating different accident theoretical reflections with investigatory practice.

## Introduction

When accidents happen, we search for a cause: why did it happen; who is responsible; what is the root cause; was it neglect; could it be avoided? Accident models and theoretical foundations underpinning safety investigations are key to understanding how investigators construct causality and make recommendations [1]. Safety science has devoted large efforts to investigating and theorizing about accidents and informing the patient safety field in the 20 years following the To Err is Human report [2], by focusing on culture, learning and systems perspectives [3–4]. So why doesn’t healthcare pay more attention to these theories when investigating healthcare accidents, more specifically? Established accident theories can support safety investigations in healthcare and provide new lenses to investigatory bodies. Here, we discuss how.

### Understanding accidents from safety science perspectives

Since the 1930s, different schools in safety science [1,5–6] have grappled with understanding the accident fundamentals and the construction of causation. Healthcare, however, remains stuck with linear models, often conceptualizing accidents as chains of events triggered by a human or mechanical root cause [1,7]. Despite modern investigatory practices in other industries, see Canham *et al*. [8], previous research has indicated that healthcare investigations consistently construed accident causation as ‘deviation from the norm in the event’s immediate temporal and spatial proximity’ [1:p.75]. This means investigations looked at why people close to the accident did not follow procedures—reasoning that if they did, it would not have happened. This immature conceptualization of causality hampers the utility of current healthcare investigations.

**Table 1:** Selected safety science schools of thought and relevance for healthcare safety investigations

Perspectives	Relevance for healthcare safety investigations
Energy and barriers	**Individual, technical, cultural and organizational barriers hinder accidents from developing and escalating** Questions for consideration What risks must be identified and managed? How did the organization establish barrier systems? Independent barriers? Multiple barriers? Technical barriers? How did the organization allocate responsibility to maintain and update barriers?
Normal accidents	**Accidents are normal, will happen and are caused by the complexity of the system** Questions for consideration Was the system functionally designed to handle its risks? Tight or loose couplings? Linear or complex interactions? Centralized or decentralized decision-making? Base on your findings Do procedures fit this system–or does it need flexibility and distributed decision-making?
High reliability	**Organizing for safety is first priority** Questions for consideration How did the organizations focus on creating redundancy, safety culture and learning mechanisms? To what degree was decision-making distributed to people with expertise? To what degree did the organization have overlapping competence, personnel and perspectives to understand and handle risks? What kind of training philosophy and practice existed?
Information management	**Information is key to understand and learn from accidents, and requires information management mechanisms** Questions for consideration What were the cultural beliefs and assumptions in the organization? What kind of warning signals had been raised? How easy was it to raise warnings? What mechanisms existed to report and learn from adverse events? Did leaders welcome critical input? What was the status of power balance, hierarchies, information sharing deficiencies over years? Accidents usually incubate over time–How was it possible?
Decision-making	**There are always numerous and simultaneous priorities. Risk and safety need to be balanced in goal conflicts with productivity and efficiency demands** Questions for consideration What kind of internal and external pressure existed on staff, managers and regulators? Financial demands? Change processes? Work-load demands? Were safety margins at risk? Any help or hinder from external demands, stakeholders, and environmental conditions? Safety is a multi-level phenomena Who were the key stakeholders at micro-, meso- and macro-level? How did stakeholders’ decisions influence risk? How was risk managed in organizational interfaces? How did the organization handle change processes, reforms and implementation processes?
Resilience engineering	**Focus on daily work practice. Study how work normally goes well and use it as a basis for understanding why it sometimes fail** Questions for consideration What are the key functions for normal work practice? How is work usually done? What key constraints must be in place? (time, resources and competence) What systems help monitor and inform work performance? To what degree is adaptive capacity important for the work? Was adaptive capacity considered positive or negative for safety?

From the early linear accident models focusing on ‘energy’ and *‘*barriers’, safety science moved on to searching for causation in: how organizations seek information and learn, complexity and organizational design, culture and training, decision-making and in adaptive capacities (see Table 1). Turner’s Man-Made Disaster theory proposes that accidents develop during incubation periods with a cultural belief that the organizations operate safely. In the aftermath, ‘information management’ deficiencies mean early warning signals were ignored or missed [9]. The *‘*Normal accident theory’ targets system complexity and investigates if high-risk systems (e.g. nuclear power) are designed with linear or complex interactions, and if they are loosely or tightly coupled. These system characteristics are essential because they determine whether procedures can play a key role and whether control mechanisms can be decentralized [10]. ‘High reliability theory’ [11] investigates how organizations operating hazardous systems (e.g. aviation control, aircraft carriers) achieve high levels of reliability. Reasons for this include overlap in competence, tasks and responsibility; room for information exchange, testing and training; and requisite variety allowing for diverse perspectives. Rasmussen’s modelling of risk in a dynamic society [12] focuses on decision-making in sociotechnical systems. In a multi-level system, decisions made by policy makers and managers will affect shop floor operations. Here accident causality is found at different system levels, in adaptations to external pressure and technological changes, and in decision-makers’ effort to optimize decisions without understanding the implications on others’ performance*. ‘*Resilience engineering’ (denoted ‘Safety-II’ in later years as the field evolved, see Braithwaite [7]) focuses attention on complexity, adaptive capacity, and how high-risk systems continue to operate despite variability [13]. Adaptations to stress and disruption are fundamental. Inspired by this school, investigators need to understand everyday work as a foundation to understand why it goes wrong: what are the key functions, resources, time and constraints, and are current procedures relevant? To expand its current vision, healthcare safety investigations can learn from these safety science schools of thought [6] (Table 1).

### Suggestions for healthcare investigations

Healthcare investigations often apply root cause analysis (RCA), to determine contributing factors and to develop solutions, but evidence shows that RCA has practical problems, e.g. lack of safety expertise in the teams, reliance on a linear conceptualization of accidents, inability to meet strict timelines, lack of independence and challenges in developing and implementing recommendations [8, 14,15]. Other identified practical RCA problems relate to, e.g. teams focusing on the first identified causal factor, such as not following a procedure, rather than assessing these factors in a holistic way in the sociotechnical system where the event happened [16,17]. Further practical problems include adopting an overly short time perspective, and using only interviews and meetings as main data collection methods thereby hampering the identification of the contributing causal factors in the working environment, in the work flow and in the wider organizational context [16,18]. In terms of problems related to identifying solutions, literature suggests that these often focus on fixing individuals instead of the system—despite being a method for system level improvement. In a review of 302 RCA’s, Kellogg and colleagues demonstrated that most solutions were related to training, process change and policy reinforcement. These solutions were characterized as weak [16,17]. Individual corrective actions are considered weaker than solutions targeting the system level [14,16].

Current thinking in healthcare investigations [15], unduly focused on these root causes and linearity, would benefit from integrating reflections and ideas from this wider literature. Accident theories can work as: different ‘accident world-view’ lenses; boundary objects for system thinking; generators for reflections and alternative explanations; and guide what to look for, recommend and learn from [1,6,19]. More specifically, the accident theories listed in Table 1 can all contribute to guide healthcare safety investigations in all phases of the investigation both in the initial searching for contributing factors by identifying questions, by searching for causality over a longer time perspective, and by searching for wider system problems; but also in directing improvement solutions as these need a system orientation. There is no single recipe for success, our argument is that we need more experimentation and use of these ideas.

Healthcare investigation practice could therefore experiment more with variety in theoretical and methodological approaches to strengthen investigation quality. For example, Canham *et al*. [8] compared a RCA investigation with a Systems-Theoretic Accident Model and Processes (STAMP) investigation of the same medication error incident of an insulin overdose from a prescription error, and developed a richer understanding of the incident as a result. The STAMP-oriented investigation focused on stakeholders and system design aspects while the RCA had more individual-based orientation of preventive actions [8]. To develop a more broad understanding of factors associated with accidents, we suggest in Table 1 how healthcare investigators could reflect and integrate safety science ideas and theories in their investigatory practice. This doesn’t mean jettisoning current investigation practices, but involves raising new questions, questioning initial approaches and underlying assumptions, and looking more towards system level factors and interaction across system interfaces. We have listed questions for this purpose in Table 1 as suggestions, this could be enriched, enlarged and improved by additional questions, reflections and possible solutions.

Experimenting with methodological approaches derived from the accident theories, such as functional resonance analysis method based on Hollnagel’s resilience thinking and ACCIMAP based on Rasmussen’s modelling of risk can also expand the understanding of what happened and drive development of more effective solutions [20]. In line with recent advances in patient safety thinking [3,4], we also suggest healthcare investigation bodies integrate competence, not only in human factors (a common recommendation), but also draw more on the wider safety science field where organizational learning and design, system dynamics, risk analysis, modelling and management are fundamental. Safety science is not learned in a 2-Day RCA course; these theories are integral to professional training.

## Conclusion

Healthcare safety investigations and reports serve multiple purposes [21]. There is untapped improvement potential. We encourage experimenting with integrating plurality in accident theoretical reflections and methods into investigatory practice. Safety science is a dynamic field, under-used in healthcare safety investigations. It’s time to step it up.

## References

[1] Wrigstad J, Bergström J, Gustafson P One event, three investigations: the reproduction of a safety norm. Saf Sci 2017;96:75–83.

[2] Kohn LT, Corrigan JM, Donaldson MS To Err is Human: Building a Safer Health System. Institute of Medicine (US) Committee on Quality of Health Care in America Washington, DC: National Academy Press, 2000. 25077248

[3] Wears R, Sutcliffe K Still Not Safe. Patient Safety and the Middle-Managing of American medicine. New York, NY: Oxford University Press, 2020.

[4] Pronovost P, Ravitz AD, Stoll RA et al. Transforming Patient Safety: A Sector-Wide Systems Approach. Report of the WISH patient safety forum 2015 World Innovation Summit for Health, Qatar Foundation, 2015.

[5] Hale A Foundations of safety science: a postscript. Saf Sci 2014;67:64–9.

[6] Rosness R, Grøtan TO, Guttormsen G et al. Organizational accidents and Resilient Organizations: Six Perspectives. Revison 2. Sintef Technology and Society: Trondheim, 2010.

[7] Braithwaite J, Wear R, Hollnagel E Resilient health care: turning patient safety on its head. Int J Qual Health Care 2015;27:418–20. 26294709 10.1093/intqhc/mzv063

[8] Canham A, Jun G, Waterson P et al. Integrating systemic accident analysis into patient safety incident investigation practices. Appl Ergon 2018;72:1–9. 29885719 10.1016/j.apergo.2018.04.012

[9] Turner B, Pidgeon N Man-Made Disasters. Oxford: Butterworth-Heineman, 1997.

[10] Perrow C Normal Accidents: Living with High-Risk Technologies. Princeton: Princeton University Press, 1984.

[11] LaPorte T, Consolini P Working in practice but not in theory: theoretical challenges of "high-reliability organizations". J Public Adm Res Theory 1991;1:19–48.

[12] Rasmussen J Risk management in a dynamic society: a modelling problem. Saf Sci 1997;27:183–213.

[13] Hollnagel E, Woods DD, Leveson N (eds). Resilience Engineering. Concepts and Precepts. Hampshire: Ashgate, 2006.

[14] Nicolini D, Waring J, Mengis J Policy and practice in the use of root cause analysis to investigate clinical adverse events: mind the gap. Soc Sci Med 2011;73:217–25. 21683494 10.1016/j.socscimed.2011.05.010

[15] Peerally MF, Carr S, Waring J et al. The problem with root cause analysis. BMJ Qual Saf 2017;26:417–22. 10.1136/bmjqs-2016-005511 PMC5530340 27340202

[16] Trbovich P, Shojania KG Root-cause analysis: swatting at mosquitoes versus draining the swamp. BMJ Qual Saf 2017;26:350–3. 10.1136/bmjqs-2016-006229 28228469

[17] Kellogg KM, Hettinger Z, Shah M et al. Our current approach to root cause analysis: is it contributing to our failure to improve patient safety? BMJ Qual Saf 2017;26:381–7. 10.1136/bmjqs-2016-005991 27940638

[18] Vincent C, Carthey J, Macrae C et al. Safety analysis over time: seven major changes to adverse event investigation. Imp Sci 12:151. 10.1186/s13012-017-0695-4 PMC5745912 29282080

[19] Wiig S, Robert G, Anderson J et al. Applying different quality and safety models in healthcare improvement work: boundary objects and system thinking. Reliabil Eng Syst Saf 2014;125:134–44.

[20] Hulme A, Stanton NA, Walker GH et al. What do applications of systems thinking accident analysis methods tell us about accident causation? A systematic review of applications between 1990 and 2018. Saf Sci 2019;117:164–83.

[21] Behr L, Grit K, Bal R et al. Framing and reframing critical incidents in hospitals. Health Risk Soc 2015;17:81–97.

